# Current status and trends in forest genomics

**DOI:** 10.48130/FR-2022-0011

**Published:** 2022-08-31

**Authors:** Dulal Borthakur, Victor Busov, Xuan Hieu Cao, Qingzhang Du, Oliver Gailing, Fikret Isik, Jae-Heung Ko, Chenghao Li, Quanzi Li, Shihui Niu, Guanzheng Qu, Thi Ha Giang Vu, Xiao-Ru Wang, Zhigang Wei, Lin Zhang, Hairong Wei

**Affiliations:** 1 Dulal Borthakur, Department of Molecular Biosciences and Bioengineering, University of Hawaii at Manoa, 1955 East-West Road, Honolulu, HI 96822, USA; 2 College of Forest Resources and Environmental Science, Michigan Technological University, Houghton, MI 49931, USA; 3 Forest Genetics and Forest Tree Breeding, Faculty for Forest Sciences and Forest Ecology, University of Göttingen, Büsgenweg 2, 37077 Göttingen, Germany; 4 National Engineering Research Center of Tree Breeding and Ecological Restoration, College of Biological Sciences and Technology, Beijing Forestry University, Beijing 100083, P.R. China; 5 Cooperative Tree Improvement Program, North Carolina State University, Raleigh, NC 27695, USA; 6 Department of Plant & Environmental New Resources, Kyung Hee University, 1732 Deogyeong-daero, Yongin 17104, Republic of Korea; 7 State Key Laboratory of Tree Genetics and Breeding, Northeast Forestry University, Harbin 150040, P.R. China; 8 State Key Laboratory of Tree Genetics and Breeding, Chinese Academy of Forestry, Beijing 100093, P.R. China; 9 Department of Ecology and Environmental Science, Umeå Plant Science Centre, Umeå University, Umeå 90187, Sweden; 10 College of Life Sciences, Heilongjiang University, Harbin 150080, P. R. China; 11 Key Laboratory of Cultivation and Protection for Non-Wood Forest Trees, Ministry of Education, Central South University of Forestry and Technology, Changsha 410004, Hunan Province, P.R. China

**Keywords:** Wood formation, Single cell RNA-seq, CRISPR-mediated genome editing, Haploid induction, Genome assembly, Perennial growth and seasonality regulation, Forest genetic diversity and climate adaption, Transformation and regeneration, QTL, association Studies, and genomic selection, Systems biology and data analysis

## Abstract

Forests are not only the most predominant of the Earth's terrestrial ecosystems, but are also the core supply for essential products for human use. However, global climate change and ongoing population explosion severely threatens the health of the forest ecosystem and aggravtes the deforestation and forest degradation. Forest genomics has great potential of increasing forest productivity and adaptation to the changing climate. In the last two decades, the field of forest genomics has advanced quickly owing to the advent of multiple high-throughput sequencing technologies, single cell RNA-seq, clustered regularly interspaced short palindromic repeats (CRISPR)-mediated genome editing, and spatial transcriptomes, as well as bioinformatics analysis technologies, which have led to the generation of multidimensional, multilayered, and spatiotemporal gene expression data. These technologies, together with basic technologies routinely used in plant biotechnology, enable us to tackle many important or unique issues in forest biology, and provide a panoramic view and an integrative elucidation of molecular regulatory mechanisms underlying phenotypic changes and variations. In this review, we recapitulated the advancement and current status of 12 research branches of forest genomics, and then provided future research directions and focuses for each area. Evidently, a shift from simple biotechnology-based research to advanced and integrative genomics research, and a setup for investigation and interpretation of many spatiotemporal development and differentiation issues in forest genomics have just begun to emerge.

## Introduction

Forests are the predominant terrestrial component on Earth, and account for nearly three quarters of the total primary production of the Earth's biosphere. Global forests generate an estimated 21.9, 8.1 and 2.6 gigatonnes of net biomass per year by tropical, temperate and boreal forests, respectively^[1]^. Forests can absorb greenhouse gases, mainly carbon dioxide (CO_2_), and sequester carbon into wood for long-term storage. Therefore, they are essential for the Earth's ecosystem and our living planet by carrying out air purification, ground water recharge, and climate abnormality control. In addition, forests play a leading role in the global cycling of energy, carbon, water and nutrients^[2]^. However, global climate change, or the long-term shift in temperatures and weather patterns, caused primarily by the planet’s overall rising temperature since the pre-industrial period due to the increasing human activities like fossil fuel burning, threatens the health of forests.

Forests are also the core raw sources for many bioproducts, which include timber, pulp, fuel wood, feedstock, and a variety of traditional and novel bioproducts. As the world population is projected to reach 9−10 billion by 2050, demand for forest-based products will continue to increase rapidly, therefore, the pressure to protect forests from deforestation and destruction are mounting up, intensifying the challenge of maintaining the health of the Earth's biosphere.

One of the strategies to counter the aforementioned challenges is to increase forest productivity, adaptation, resilience and sustainability, which requires the development of multifaced approaches where forest genomics play a key role^[3]^. Firstly, genomics-based approaches can significantly increase the productivity and adaptation directly through modification of one or multiple genes in tree genomes. Secondly, genomic-based approaches are particularly instrumental for identifying the genetic markers and genes for gene pyramiding in molecular breeding. Therefore, significantly enhancing the efficiency of conventional breeding in targeting the complex and low heritable quantitative traits of interest in forest trees. Thirdly, genomic approaches have great potential to accelerate the generation of system biology knowledge that are essential for determination of the optimal strategies and plans for improving forests. Fourthly, computational genomics provides an alternate approach to identify genes regulating the complex traits, enabling gene stacking and genome editing for developing custom-designed trees for designated uses. Finally, genomics-based approaches can answer many unique biological questions of forest trees, including their unusual reproductive features, woody and perennial growth habit, and their mechanisms of adaptation to abiotic and biotic stress^[4]^.

In this review, we summarized the status, progress and future directions of multiple facets of forest genomics research, and hope this review can aid and foster the developments in genomics to enhance the productivity and sustainability, and accelerate the domestication of forest trees for combating climate change and meeting the increasing demands for forest products caused by the continue explosion of the World's population.

## Wood formation

Environmentally friendly, renewable and sustainable woody biomass is needed to save humanity from the global climate crisis. Woody biomass, or xylem cells with thickened secondary cell walls (SCW), composed of cellulose, hemicellulose (xylan) and lignin (for a recent review, see Schultz et al.^[5]^), accounts for about 90% of the total biomass produced on Earth^[6,7]^. Cellulose and xylan are polymers of glucose and xylose, respectively, and lignin is a polymer of phenolic compounds. These three types of polymers can be utilized to produce not only biofuels but also various traditional and new materials, such as, bioplastics, carbon nanomaterials, pharmaceuticals, and various synthetic materials by using appropriate conversion technology (for a recent review, see de Vries et al.^[8]^). Therefore, the utilization of woody biomass can not only partially replace energy and materials provided by existing fossil fuels, but also reduce atmospheric carbon dioxide to mitigate the global climate crisis.

Wood is formed by complex but highly organized regulatory processes that include vascular cambium cell division and development, and xylem cell differentiation. Extensive studies have been performed to resolve molecular networks controlling vascular cambium development and xylem cell differentiation, mainly using plant species such as *Arabidopsis*, *Populus, Eucalyptus, Zinnia elegance*, and other species^[9−11]^. For xylem cell differentiation, vascular-related NAC domain (VND) subfamily proteins, VND6 and VND7, which are identified as master regulators for metaxylem (i.e., SCW deposited with pitted or reticular form) and protoxylem (i.e., SCW with helical form) differentiation, respectively^[10]^, regulate transcription of SCW forming genes (e.g., MYB46 and MYB83, a paralog of MYB46) and programmed cell death (PCD)^[12,13]^. MYB46 and MYB83, the master regulators of SCW biosynthesis found in *Arabidopsis*, regulate directly/indirectly downstream transcription factors and structural SCW biosynthesis genes in a feed-forward manner through a highly complex and sophisticated regulatory network^[12,13]^. Recently, it was reported that PtrHB7, a member of HD-ZIP III family in poplar, is involved in the auxin-induced xylem differentiation regulatory network in woody stems^[14]^.

Cylindrical vascular cambium is a secondary meristem containing bifacial stem cells^[15,16]^. Recently, considerable progress has been made in the molecular understanding of the development of vascular cambium, and it has been revealed that the coordinative regulatory mechanisms, including transcription factors, peptides and hormones, are all required for this process^[13,17]^. Both auxin and cytokinin (CK) play pivotal roles in regulating the initiation and maintenance of procambial cells. Auxin activates MONOPTEROS (MP), which positively regulates vascular initial cell divisions through auxin efflux carrier *PIN-FORMED1* (*PIN1*) and TARGET OF MONOPTEROS 5 (TMO5)/LONESOME HIGHWAY (LHW) dimer^[18−20]^. LONELY GUY 4 (LOG4), a rate-limiting enzyme in CK biosynthesis, is directly activated by the TMO5/LHW dimer, and the resulting CK promotes cell divisions through the DOF transcription factor, DOF2.1^[19,21]^. It has been known that the auxin-signaling IAA12-ARF5/MP module controls *Arabidopsis* provascular specification and patterning^[22]^. A similar module (PtoIAA9-PtoARF5) was recently found to operate in auxin-triggered xylem cell differentiation in *Populus*^[14]^. In addition to plant hormones, TRACHEARY ELEMENT DIFFERENTIATION INHIBITORY FACTOR (TDIF)-PHLOEM INTERCALATED WITH XYLEM (PXY)-WUSCHEL-RELATED HOMEOBOX (WOX) signaling module regulates cambial cell division and radial growth in trees (for a recent review see Wang et al.^[11]^).

Currently, the understanding of the underlying molecular mechanisms by which the procambium develops into the vascular cambium is still limited. In this process, the parenchymatous cells in the interfascicular region must be differentiated into cambium to connect the fascicular cambium. Recently, the HD-ZIP III gene *PtrHB4* was found to be involved in the induction of interfascicular cambial cell division in *Populus* stem^[13]^. Therefore, further analysis of target genes directly downstream of PtHB4 is expected to provide clues to this process.

Future research for wood formation may be focused on the cambium cell identity maintenance, and development and differentiation process of cambium cells into secondary xylem and phloem by combining sc-RNA-seq and spatiotemporal transcriptome-based technologies, which allows the determination of new cell- and tissue-specific regulators during these processes. Single cell RNA sequencing (scRNA-seq) has recently been used to investigate vascular cell specification and differentiation in trees at single-cell resolution using poplar stems^[23,24]^. The advent of spatiotemporal transcriptome technologies, for example, stereo-seq using nanoball array^[25]^, will enable the mapping of spatiotemporal transcriptomic dynamics and changes during the formation of secondary xylem and phloem, which will significantly advance our understanding of this important process.

## CRISPR-mediated genome editing provides a powerful tool for forest tree improvement

With its versatility, high efficiency, and robustness, Clustered Regularly Interspaced Short Palindromic Repeats (CRISPR) biotechnology has emerged as the most widely used genome editing tool. To date, SRISPR-based technologies have been harnessed to introduce precise alterations at the target sites in many given plant species. For recent reviews in forest trees, see^[26,27]^).

Genome editing and genetic manipulation of forest trees have become leading edge research fields. Several types of the engineered Cas9s have been utilized to establish the CRISPR/Cas9 application in several forest trees^[28−34]^. Among these, poplar and eucalypt genomes are the most common and successful genomes that have been subjected to genome editing due to well-established *Agrobacterium*-mediated stable transformation and abundant genetic and genomic resources and tools. Nevertheless, there is substantial room for the improvement of specificity and efficiency in CRISPR-edited tree experiments, given that the CRISPR toolbox for plant genome editing has been continuously expanded with unexplored or newly engineered CRISPR effectors (for reviews, see^[35−37]^). In addition, versatile methods for targeted insertion or replacement of genes have been demonstrated to improve the efficiency of HDR-mediated precise modification, such as tandem repeat-HDR^[38]^ and transcript-templated HDR^[39]^. Morever, genome editing approaches without introducing DSBs via a variety of CRISPR-mediated base editing^[40]^ and prime editing methods have recently been developed^[41]^. The first cytosine base editor for conversions of target cytosines to thymines was designed by the fusion of a nickase Cas9 and a cytidine deaminase enzyme^[40,42]^.

In general, CRISPR-mediated genome editing holds great potential for genetic engineering of forest trees, studying and understanding their growth, resilience and adaptation during climate change. Several studies with genetically-modified or CRISPR-mediated improvement of wood quality^[43]^, resistance to pests and diseases^[44]^, tolerance to drought^[45]^, salt and cold stresses^[46]^ have been conducted in poplar, black locust^[47]^, mulberries^[48]^, and upland cotton trees^[46]^ (for recent reviews, see^[26,49,50]^). We anticipate that many more CRISPR-based applications and studies will be performed in the following areas. Firstly, biological functions of candidate genes from quantitative trait loci (QTL) and association studies or homologous genes of interest from other plant species can be studied in loss-of-function mutants. Secondly, functions of allelic variants even with small effects in association studies can be investigated by precise knock-in CRISPR systems (i.e., HDR-mediated gene replacement, base editing, and prime editing). Thirdly, improvement of quantitative traits can be achieved by simultaneously inserting or exchanging different regulatory elements (i.e., enhancers and promoters) from functionally known genes, modulating the expression levels of genes in the known gene regulatory network as well as fine-tuning biological pathway switches and rate-limiting enzymes. Lastly, gain-of-function CRISPRed trees with better resistance to disease^[44]^ and adaptation enhanced traits can be pursued by CRISPR-based transcriptional activation of endogenous genes. Altogether, the robustness of the CRISPR technology allows researchers in the field of forest genetics and forest tree breeding to take advantage of innovative ideas from all plant genome editing projects such as climate-resilient crops and fruit trees. With the significant and rapid progress in CRISPR technologies, a new green revolution in forest tree breeding and conservation with more climate resilient forests might become reality in the near future.

More recently, new precise DNA base substitutions have been established (for a review, see^[51]^), including A–G base transition^[52]^, C–A^[53]^, and C–G transversion^[53−55]^. Importantly, using a nickase Cas9 fused to a reverse-transcriptase (RT), the prime-editing tools^[41]^ can mediate desired combinations of deletions, insertions, and base-to-base conversions to replace the target sequence. The RT is guided by a prime-editing guide RNA (pegRNA) that is made up of an extended gRNA specifying the target site and a RT template sequence containing desired edits. First successful demonstrations of base editing and prime editing in plant models offer great potential to solve practical problems for tree breeding by introducing novel quantitative traits with a gain-of-function mutation^[56,57]^. However, great challenges remain because many attractive target traits for tree improvement including tree growth, wood properties, stress and disease resistance are highly polygenic and under complex regulation networks. CRISPR-based gene manipulations can possibly generate unintended consequences due to, for example, imbalances in the gene interaction networks. These limitations might be mitigated by CRISPR-based pyramiding of multiple monogenic traits or polygenic traits, taking advantage of the robustness, precision and multiplexity of the CRISPR technology in combination with genetic crosses and marker-assisted selection. Nevertheless, a wider application of CRISPR-mediated gene editing in forestry requests a significant improvement of the low efficiency of the CRISPR-based editing technologies and to overcome the recalcitrance in transformation and regeneration of many tree species.

## Single-cell RNA-sequencing technology and implication in forest genomics

The development of multicellular plants involves the formation of organs that are comprised of different types of cells with high heterogeneity. During organ formation, cell fate determination and differentiation are precisely controlled by successive transcriptional regulations. However, the cell fate determination from the cambium to the secondary xylem or secondary phloem also remains elusive. Since the existing transcriptome analysis (RNA-seq) can only obtain information on a whole tissue, it is difficult to distinguish and analyze different cell types. With the successful isolation of single cells, scRNA-seq has become a powerful tool to study the gene expressions for individual cells among a heterogenous tissue. Because it can classify cells into different groups based on their types and quantify cell type-specific expression, and also enables cell trajectory analysis for cell differentiation and development. Thus, by reconstructing the spatiotemporal relationship between the cambium and surrounding cells, it is possible to track the progressive cell fate changes of secondary xylem and phloem, respectively, and to uncover new key cell type-specific regulators.

Single cells can be separated by using limiting dilution, micromanipulation, laser capture microdissection, flow cytometry and microfluidics^[58]^. In existing scRNA-seq experimental procedures, thousands of individual cells, upon being isolated with droplet-based microfluidics^[59]^, can be barcoded to distinguish transcripts from different cells, allowing a high throughput gene expression profiling at single-cell resolution^[60−62]^. Based on the differential gene expression patterns, these cells can be grouped into different clusters, which can be classified to specific cell types. The analysis of cell clusters enables to understand the cell heterogeneity and provides additional markers for each cell type^[60]^. Since the cells that are undergoing the transition from one to another state could be captured, novel cell types may be uncovered, and using pseudo-time analysis to make the order between single cells based on the similarity of gene expression patterns could deduce the developmental trajectories of the clusters (cell types). New regulators controlling developmental transition can be identified, improving the investigation of dynamic developmental processes.

Microfluidic-based scRNA-seq was first applied in *Arabidopsis* roots and showed the feasibility of high throughput scRNA-seq in plants^[63]^. Additional scRNA-seq studies in *Arabidopsis* roots used either drop-seq^[64−67]^ or 10X genomics methods^[68]^ to provide detailed spatiotemporal information for different cell types, including the quiescent center that has stem cells, and reconstruct the continuous differentiation trajectory of root epidermal, endodermical root hair cells, and other cell types. scRNA-seq was also applied to rice roots^[69,70]^ and other tissues of plants, including shoot tips, lateral roots, gametophytes, anthers, etc.^[71−81]^. Compared to these model plant species, the application of scRNA-seq in trees is limited. The first scRNA-seq was performed in the differentiating xylem of *Populus alba* × *Populus glandulosa*^[24]^. This study profiled 9798 cells and identified 12 cell clusters, encompassing vessel cells, fiber cells, ray parenchyma cells, and xylem precursor cells. Further pseudo-time analysis revealed the differentiating trajectory of fiber cells, ray parenchyma cells and vessels. Chen et al.^[23]^. performed scRNA-seq on protoplasts harvested separately from *P. alba* var. *pyramidalis* stem bark and wood. This study also identified the marker genes for the phloem, and reconstructed the cell differentiation trajectories for phloem and xylem development from cambium. The scRNA-seq technique was very recently used to investigate vascular cell specification and differentiation in trees at single-cell resolution using poplar stems^[23,24]^. Compared to spatial transcriptome technology like Stereo-seq, the information we can obtain from scRNA-seq is still very limited.

Most of the current studies in plants used protoplasts for scRNA-seq, which relies on successful protoplast preparation. It is challenging to isolate high quality protoplasts from the cells that are located in the inner tissue of an organ and have thick cell walls, especially in trees. High protoplast preparation efficiency needs to be improved for some specific tissues and in most species. Longer incubation times of enzymatic digestions to remove cell walls may lead to certain changes in transcriptional activity^[82]^. Single-nucleus RNA sequencing (snRNA-seq) can avoid the protoplasting effect, and snRNA-seq protocols have been established in *Arabidopsis* and rice^[83−88]^, and recently in poplar^[89]^. Although it was observed that expression of fewer genes was captured per cell by snRNA-seq than scRNA-seq, it is still worth to expand snRNA-seq for other tissues. The scRNA-seq data can be integrated with other transcriptome data to explore key regulators and novel mechanisms. For example, the integration analysis of snRNA-seq and snATAC-seq elucidated cell-type-specific patterns of chromatin accessibility for the cell-type-specific markers, showing that chromatin accessibility can be used as molecular markers to indicate root hair and endodermal cell developmental states^[83]^. Through analysis of single cell transcriptome overexpression of *VND7*, *MYB46* and *MYB8*3, Turco et al. identified that other four targets of VND7, but not MYB46 and MYB83, were involved in VND7-mediated switch of root cells to xylem cell identity^[90]^. Further wide applications of scRNA-seq in mutants or transgenics will enhance the understanding on the transcriptional regulation of a gene of interest. However, scRNA-seq lacks spatial information, which can be overcome by combination with the spatial transcriptome^[91,92]^, and this combination has not been reported in plants. Overall, increasing number of scRNA-seq studies have been performed in plants and provide insights into the transcriptional regulation of cellular state during organ formation. It is anticipated that other analyses, including chromatin immunoprecipitation (ChIP)-seq, DNA methylation, protein-protein interactions at single-cell level, will also be achieved successfully in plants in the near future. The limiting factors for implementation of scRNA-seq to forest trees include the challenges in target tissue isolation and removal of cell walls. Compared to other plant species, both target tissue isolation and cell wall removal in woody plants are more challenging due to rigidness, and lignified and thickened cell walls.

## Haploid induction and breeding

High-levels of heterozygosity are a significant obstacle for not only the genome sequencing and assembly but also tree breeding through inbred lines, which can be used to generate heterosis through crossing. Although the endosperms originated from female gametophyte tissue (n) in gymnosperms seeds and some angiosperm seeds are haploid, the haploid tissue in a single seed is generally insufficient for extracting enough DNA for haploid genome sequencing. Generating inbred lines for creating heterosis through cross-hybridization takes at least six generations to achieve approximate complete homozygosity in most agricultural crops. This can take even more generations and longer time for tree species due to their higher levels of heterozygosity, long juvenile phases, and long-generation cycles as well as self-incompatibility. Therefore, it is practically impossible to implement inbreeding improvement strategy in trees in the conventional manner. A strategy to overcome these obstacles is to obtain haploid plants, which can not only facilitate genome sequencing and assembly but also generate homozygosity through chromosomal doubling. Doubled haploid (DH) lines are even better than inbred lines, and thus can be of great value for tree breeding, genome sequencing and assembly, and also other genome-based research.

For agricultural crops, haploids can be induced in natural conditions or artificially by a physical or chemical treatment. Spontaneous haploids occurred in many species including maize, cotton, rice tomato, barley, and brassica^[93]^. However, the induction rates are very low in many species, and as a result, selection of spontaneous haploids from natural populations is not cost-effective. Subsequently, numerous endeavors have been made to increase the frequency of artificial haploid induction. It has been found that a specific maternal or paternal parent can give rise to a higher induction frequency. For example, a maize inbred designated as 'stock 6' could generate 3.23% haploids in 10,616 progenies^[94]^. Hence, selection of parents that have higher haploid induction frequency was effective and accelerated maize inbreed line generation. To date, various techniques have been employed in different plant species, some of which have been reviewed in several reviews^[95−97]^. One of these technique is distant pollination, namely wide crossing, which is one of the most effective methods for female-derived haploid production. For example, haploid plants were obtained from *Lactuca sativa* pollinated with the pollen from distant species like *Helianthus annuus* and *H. tuberosus*^[98]^. The pollen tubes of the distant species may not release the sperm cells into the ovaries but can stimulate the development of ovaries into haploid seeds. In addition, the female-derived haploid induction by pollination with the pollen pretreated with various physical or chemical agents has also been used in many species. For example, haploid plants were obtained by pollination with irradiated pollen in melon^[99,100]^. In addition, gynogenesis induction by direct *in vitro* culture of unfertilized ovaries or ovules is another viable approach for haploid production for some species, such as cucumber and red beet^[101,102]^. Moreover, androgenesis induction by anther culture is one of the more frequently used methods in various plant species, such as wheat^[103]^, cucurbita^[104]^; *Cannabis sativa*^[105]^. Those methods that were successfully used in agricultural crops need to be tested for trees which are mostly self-incompatible.

To date, some aforementioned approaches have been adapted or modified to produce haploids in forest trees. For example, cross-pollination with stress-treated pollen that stimulate parthenogenesis for inducing haploid trees; the pollen grains are often pretreated with physical stresses like high temperature^[106]^, heat^[107]^, irradiation^[108]^, or chemical like toluidine blue^[109]^. In addition, the unpollinated ovaries were reported to develop into haploid poplar trees^[110]^, which indicates that the female gametophyte and megaspore of angiosperms can be induced *in vitro* for sporophytic development, thereby creating a new avenue to haploid breeding. Moreover, the endosperms of most gymnosperms comprise haploid cells developed directly from the female gametophyte, and thus can be used to induce haploids directly. For example, haploid calli have been generated by culturing *Taxus chinensis* endosperm^[111]^. Haploid plants of *Eucommia ulmoides* were induced *via* parthenogenesis with heat treatment on female flower buds during the developmental stage of embryo sac formation^[112]^. Anther or pollen culture coupled with plant hormone treatments under tissue culture conditions was the most commonly used method in tree species^[113−116]^. In contrast to a wide variety of crop species that have been subjected to haploid induction, much fewer haploid tree species have been generated *in vitro* because of their recalcitrance to tissue culture and regeneration. Furthermore, the DH trees generated from haploids usually show poor vitality and can hardly survive for more than a few years until they reach flowering age. To date, no DH population of tree species has been reported to be established due to poor adaptation caused by high homozygosity. In the future, more efforts should be made to increase induction efficiency, establish effective procedures that can facilitate acquisition of a large number of DH lines, and increase their survival under greenhouse and field conditions. In recent years, a CENH3-mediated haploid induction system has been established, which has the potential to be extended to tree species since CENH3 is universally present in eukaryote species^[117−119]^.

## Recalcitrance to genetic transformation and regeneration in trees

Generation of transgenic trees with new or improved desirable traits relies on genetic transformation and subsequent regeneration. Transformation of foreign genes for molecular breeding or gene/genome editing is the key to successful tree trait improvement. Various transformation protocols including *Agrobacterium*-mediated transformation, Polyethylene glycol (PEG)-mediated direct transfer and biolistic bombardment have been applied for plants^[120]^ . Among the most widely used is *Agrobacterium*-mediated T-DNA transformation which, in general, has the highest transformation efficiency^[121]^. Subsequent regeneration is an essential process to recover transgenic plants. Callus induction, *de novo* organogenesis, and somatic embryogenesis of conventional tissue culture-based regeneration protocols have been widely developed in trees^[122]^. The earliest reports on transgenic poplar and European larch trees are dated 1987^[123]^ and 1991^[124]^, respectively. Although genetic transformation and regeneration have been widely applied to many other tree species, the practice for genetic transformation and regeneration in trees is still largely restricted to model forest (poplars and *Eucalyptus*)^[125]^ and fruit trees^[121,126]^, and has been rarely applied to industrially important trees such as rubber trees^[127]^ and conifers^[128]^ because of the recalcitrance of transformation and regeneration. Thus far, the following efforts have been made to overcome recalcitrance in genetic transformation and regeneration practices.

The most widely employed approach to overcome recalcitrance is the selection of most amenable genotypes and tissues such as juvenile leaves, petioles, cotyledons^[129−133]^ and the use of phytohormones^[134,135]^ such as auxin and cytokinin. Although these approaches work for some species, they have different effects in different species and have little general applicability. For example, for Amur cork trees^[136−138]^ and willow trees^[139,140]^, even the juvenile leaves and stem explants are recalcitrant to transformation and shoot regeneration, the shoot apical meristems germinated from the mature seeds exhibit amenability to transformation and subsequent regeneration. In conifers, somatic embryogenesis from the immature zygotic embryos is the only way to regenerate plantlets^[141,142]^. The amenability of explants to genetic transformation and regeneration differs among explant types (juvenile or mature) and genotypes; regeneration capacity of explants generally declines as age increases. Different genotypes have widely exhibited distinct regenerative responses even in the same species. Loss of phytohormone responsiveness is a putative cause for declined regenerative capacity. As plants get older, the developmental stages and differentiation as well as epigenetic modifications deactivate phytohormone responsiveness, thereby resulting in different degrees of recalcitrancy^[143]^. Some genes that control transformation amenability/regeneration have been identified, e.g. *Baby boom* (*BBM*), *WUSCHEL*, *SHOOT MERISTEMLESS* (*STM*), *WOX*^[144,145]^, and the synthetic gene for the GROWTH-REGULATING FACTOR (GRF)- GRF-INTERACTING FACTOR (GIF) chimeric protein^[146]^, and these genes have been used to improve regeneration potential. Co/pre-transformation has also been reported to boost genetic transformation and regeneration in trees^[147,148]^.

In general, phytohormones including auxin, cytokinin, and abscisic acid trigger signaling events for plant regeneration which then relieve epigenetic constraints and activates the above developmental regulator genes to initiate cell-fate transitions (dedifferentiation) and expression of downstream genes for plant development - usually the hormone biosynthesis and signaling genes (redifferentiation)^[143,149,150]^. GRF-GIF chimeric protein has recently been highlighted for its capacity to remove epigenetic regulatory barriers against plant regeneration. The expression of developmental genes driving the cell transition processes for dedifferentiation and redifferentiation during plant regeneration are strictly regulated by a closed chromatin state that can be opened by epigenetic factors such as SWITCH/SUCROSE NONFERMENTING (SWI/SNF) complexes. GRF and its cofactor GIF form chimera which then can recruit SWI/SNF complexes to regeneration-related genes to remodeling chromatin structures and states, relieving the constraints for the transcription of those genes to promote regeneration^[151]^.

Owing to the above efforts, genetic transformation and regeneration have significantly improved; however, the following questions on recalcitrance remain to be answered:

(1) What are the upstream regulatory genes for the developmental genes that control transformation amenability/regeneration including *BBM*, *WUSCHEL*, *STM*, and *WOX,* and how are the developmental genes upon a phytohormone treatment triggered and regulated?

(2) What factors cause loss of phytohormone responsiveness during aging and how to revert the process?

(3) What are the key factors that determine embryogenic competence during somatic embryogenesis?

(4) How are the external signals such as stresses and phytohormones transduced to initiate upregulation of those factors during somatic embryogenesis?

(5) How does the aging or subculture affect embryogenic competence of explants?

Providing answers to these questions will significantly advance our understanding of transformation and regeneration. Moreover, conventional regeneration protocols based on tissue culture are laborious and time-consuming, especially for trees which generally have a longer generation time than crops. Some scientists have tried to bypass the regeneration process to circumvent regeneration-recalcitrant issues. For example, tissue culture-free transformation systems have been established in *Arabidopsis* and tobacco^[152,153]^. However, these novel protocols cannot yet completely bypass the tissue culture in the whole transformation process, and are not yet been applied to tree species. Overall, the efforts to overcome the recalcitrancy issue have greatly advanced the genetic transformation and regeneration in trees, but there is still a long way to go. There is little evidence that substantiates the molecular mechanism underlying recalcitrance and further work should be continued to obtain in-depth insights into the process. It will be of great significance in gene transformation and regeneration practices in trees.

## Nitrogen fixing: A special feature of *Leucaena-*Rhizobium symbiosis

Legume-*Rhizobium* symbiosis is a beneficial mutualistic interaction between legume hosts and compatible rhizobia that exhibit species specificity. Most current knowledge of legume-*Rhizobium* symbiosis has been derived from studies of nodulation and nitrogen fixation in field legumes, such as alfalfa, peas, beans, and soybean, and model legume species, such as *Lotus japonicus* and *Medicago truncatula*, through their interactions with compatible rhizobia^[154]^. The knowledge of nitrogen-fixing symbiosis between tree legumes and rhizobia is relatively scarce. Moreover, some tree legumes have uncommon features, for example, *Acacia koa*, a tree legume endemic to the Hawaiian Islands, produces both root and canopy nodulation in symbiosis with *Bradyrhizobium*^[155]^. Symbiotic nitrogen fixation in the tree-legume *Leucaena* in association with specific fast-growing rhizobia has an additional feature. Leucaena produces a toxic compound, mimosine, which is present in all parts of the plant, including roots and root nodules. Mimosine is also secreted to the rhizosphere in the root exudate^[156]^. Rhizobia in the rhizosphere or in the nodule have to overcome mimosine toxicity. Rhizobia that effectively nodulate *Leucaena* degrade mimosine in both rhizosphere and inside root nodules.

**Figure 1:**
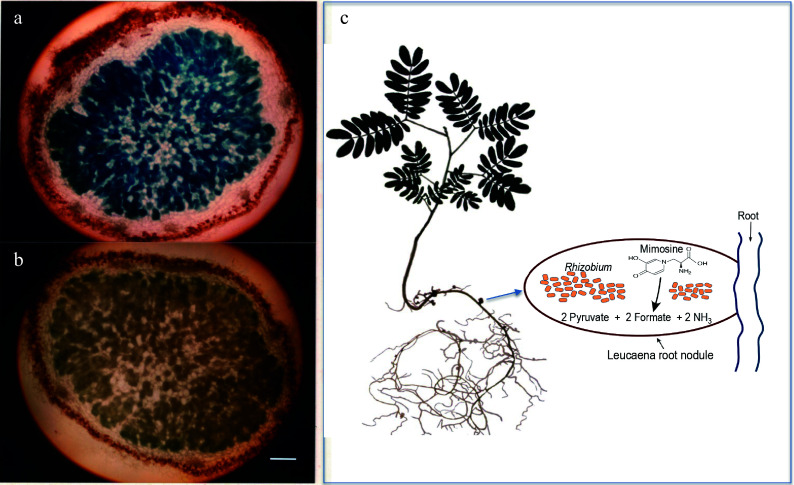
Light microscopy of 20-µM thick sections of 5-week-old *Leucaena leucocephala* nodules. (a) Induction of alkaline phosphatase activity in the *Leucaena* nodule formed by the *midA::phoA* mutant RUH128 of *Rhizobium* TAL1145. (b) Another mutant RUH129, in which the *phoA* insertion on *midA* was in the opposite orientation, was used as a negative control. Nodule sections were stained for phosphatase activity. RUH128 cells inside the nodule expressed phosphatase activity whereas RUH129 cells inside the nodule did not show any detectable phosphatase activity. Bar represents 400 µM. (c) Schematic of a *Leucaena* nodule occupied by a *Rhizobium* strain that degrades mimosine into pyruvate, formate and ammonia.

The genes for mimosine degradation have been well-characterized in *Rhizobium* sp. strain TAL1145 that effectively nodulates *Leucaena*. Mimosine degradation by *Rhizobium* takes place in two steps. First, mimosine is degraded to ammonia, pyruvate and 3-hydroxy-4-pyridone (3H4P) by a C–N lyase, called 'rhizomimosinase' encoded by the *Rhizobium midD* gene^[157,158]^. 3H4P is further degraded to ammonia, pyruvate, and formate by a dioxygenase and a hydrolase encoded by the *Rhizobium pydA* and *pydB* genes, respectively^[159]^. The *midD* gene for *rhizomimosinase* is a part of the *midABCD* operon, where *midABC* encodes three constituent proteins ​of ABC transporters: ​a periplasmic mimosine-binding protein, a permease, and the ATP-binding protein, for transporting mimosine into *Rhizobium* cytoplasm. By constructing several *midA*::*gus*, *midC*::*gus* and *midD*::*gus* fusions, it was demonstrated that the *midABCD* operon is expressed in *Rhizobium* in the nodule and also inducible by mimosine^[157]^. Similarly, by constructing *midA*::*phoA* insertion mutant derivatives of TAL1145, it was shown that the *midABCD* operon expressed in *Rhizobium* bacteroids inside the *Leucaena* root nodules (Fig. 1). The expression of the *midABCD* operon is regulated by a positive regulatory protein encoded by *midR*, which has high sequence similarity with the LysR family of positive regulators. In the rhizosphere, some amounts of mimosine may be degraded to 3H4P, which can be also taken up by *Rhizobium* using a specific ABC transporter encoded by *pydC*, *pydD*, and *pydE* genes. The *pydA*, *pydB*, *pydC*, *pydD*, and *pydE* genes are inducible by mimosine, 3H4P, and several analogs of 3H4P^[^^6^^]^.

**Figure 2:**
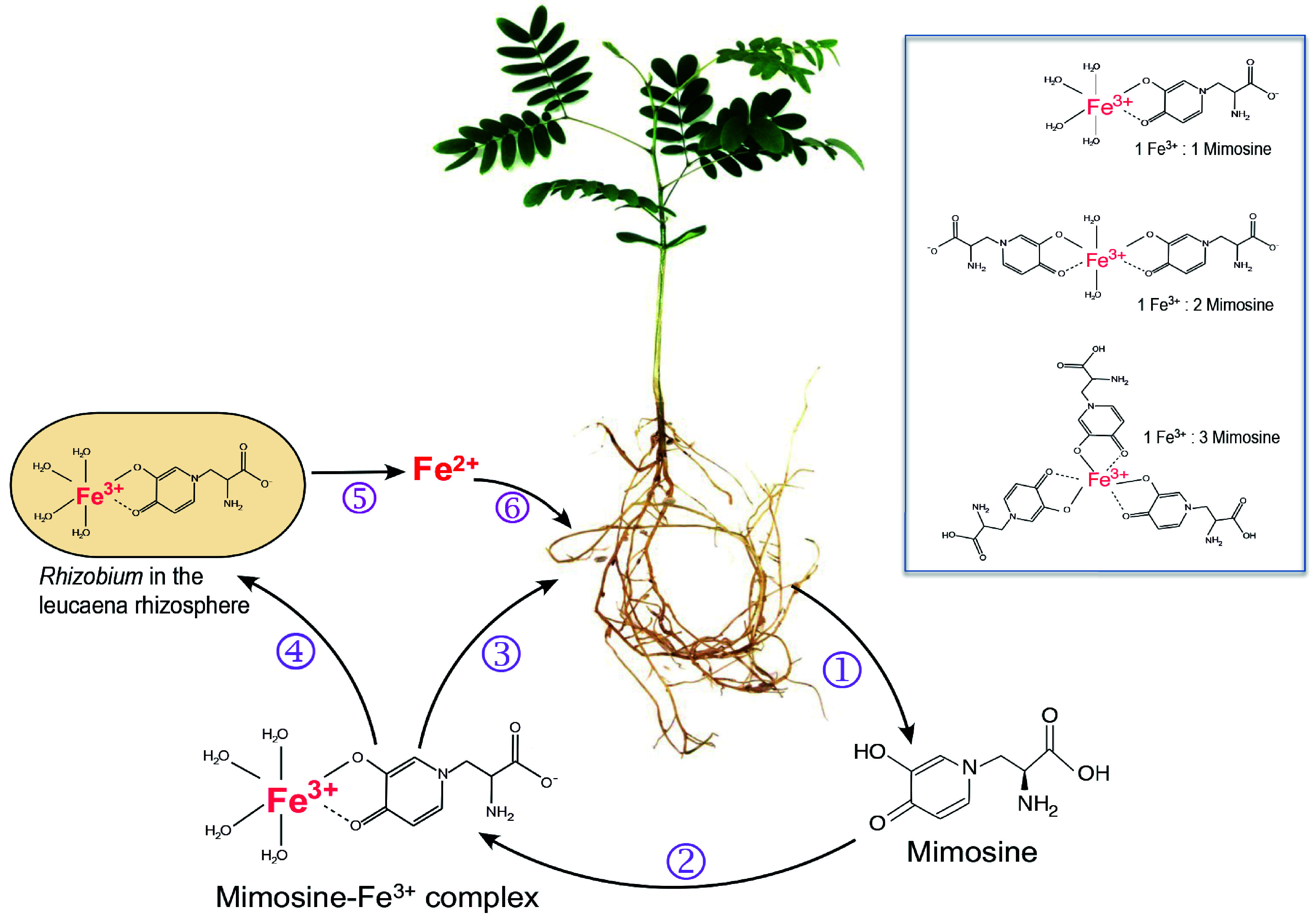
Roles of mimosine and free-living rhizobia in iron uptake by *Leucaena*. (1) Leucaena root exudates contain mimosine, which chelates Fe^3+^ to form Fe^3+^-mimosine complexes (2). Three types of Fe^3+^-mimosine complexes may be formed depending on the amount of mimosine available in the rhizosphere (box). The Fe^3+^-mimosine complexes are taken up by* Leucaena* roots using an oligopeptide-type of transporter (3). Fe^3+^-mimosine complexes can also be taken up by mimosine-degrading rhizobia in the rhizosphere using an ABC transporter (4). Rhizobia degrade Fe^3+^-mimosine complexes and release Fe^2+^ into the rhizosphere (5). Fe^2+^ is taken up by *Leucaena* through an IRT transporter (6).

The ability to degrade mimosine is not essential for forming nitrogen-fixing nodules on *Leucaena*. However, mimosine-degrading ability provides a competitive advantage for nodule occupancy and efficient nitrogen fixation in *Leucaena* nodules^[160]^. Mimosine is a bacteriostatic compound and the mimosine-non-degrading *Rhizobium* strains, such as CTAT899, do not have a mimosine-specific ABC transporter. Therefore, these rhizobia can survive and grow in the *Leucaena* rhizosphere or inside root-nodules even in the presence of mimosine. Mimosine chelates Fe^3+^ ions in the rhizosphere by forming Fe^3+^-mimosine complexes, thereby depriving these rhizobia of Fe^3+^ and reducing their growth. On the other hand, mimosine-degrading strains, such as TAL1145, can uptake Fe^3+^-mimosine complexes and utilize them as a source of carbon and nitrogen. Therefore, they can grow in the presence of mimosine and occupy nodules more effectively. In the absence of mimosine-degrading rhizobia, non-degrading rhizobia can occupy *Leucaena* nodules. About 40% of *Rhizobium* isolates obtained from the nodules of *Leucaena* growing in several locations on the island of Oahu contained mimosine-degrading rhizobia^[161]^. Our current understanding of the role of mimosine-degrading free-living rhizobia in the *Leucaena* rhizosphere is depicted in Fig. 2. Leucaena can grow successfully even in alkaline soils where iron forms insoluble hydroxides, which cannot be taken up by plants. Mimosine, secreted by *Leucaena* roots, has high binding affinity for Fe^3+^ at alkaline pH, where it forms Fe^3+^-mimosine complexes that are water soluble at alkaline pH. Depending on the availability of mimosine in the soil, three types of Fe^3+^-mimosine complexes are possible, where the ratios of Fe^3+^ and mimosine in the complexes may be 1:1, 1:2, or 1:3^[3]^ (see the box in Fig. 2). The water-soluble Fe^3+^-mimosine complexes are taken up by *Leucaena* roots through transporter proteins belonging to the oligopeptide transporter family, which includes YSL transporters. Such an iron uptake system using phytosiderophore and oligopeptide transporters is common in grasses and is known as strategy II of iron uptake. Leucaena also employs strategy I of iron uptake that involves a membrane-bound ferric chelate reductase for converting Fe^3+^ to Fe^2+^, which is then taken up by the plant through an iron-regulated transporter (IRT). Free-living rhizobia inhabiting in the *Leucaena* rhizosphere may also uptake Fe^3+^-mimosine complexes and degrade mimosine, releasing Fe^2+^ to the rhizosphere, where it is taken up by the plant using an IRT transporter (Fig. 2). Thus, besides providing a selective advantage to occupy *Leucaena* root nodules, such mimosine-degrading rhizobia inhabiting in close proximity of the Leucaena’s root system, enhance iron availability in the *Leucaena* rhizosphere. Future research will apply genome editing technology to develop new varieties of *Leucaena* for regulated expression or inhibition of the mimosine biosynthesis genes.

## Genome assembly and evolution

The publication of the genome sequence of the first tree species *Populus trichocarpa* in 2006^[162]^ marked the beginning of the genomic era of tree species. In the first decade after the poplar genome was released, a total of 47 tree species were sequenced. With the technical advantages brought by more powerful DNA sequencing technology, draft or reference genomes of 357 tree species, including 266 arboreal, 71 shrub and 20 vine species, were assembled by the end of February 2022, which accounts for about 36% of all sequenced plants.

The advantage of third-generation long-read sequencing technology is the key driving force behind the substantial improvement of genome assembly continuity in recent years. The contig N50 of published plant genome was increased from 99.5 ± 48.1 kb in 2010 to 3,395.2 ± 735.4 kb in 2020^[163]^. The Pacific Biosciences (PacBio) and Oxford Nanopore Technologies (ONT) are the two mainly long-read sequencing platforms used in the tree genome project, and to date, the adoption ratio of PacBio to ONT is five to one. The lower sequencing error rate of PacBio is the main reason for its preference, especially its recently updated circular consensus sequencing (CCS) mode which can generate highly accurate long high-fidelity (HiFi) reads^[164]^. In comparison with continuous long-read (CLR) mode, the CCS-based assembly is more efficient and time-saving. It is reported that a 27 Gb-sized hexaploidy coast redwood (*Sequoia sempervirens*) has been sequenced and assembled in only two weeks with a high contig N50 value of 1.92 Mb^[165]^. Due to its advantages, CCS will be more widely applied for tree genome sequencing, especially for giant genomes and genome phasing.

Furthermore, cost and time-effective methods as high throughput chromatin conformation capture (Hi-C)^[166]^ and BioNano optical mapping^[167]^ have greatly accelerated the scaffolding of contigs to generate chromosome-level genome assemblies, especially for forest trees in which building a high-density genetic map is generally very time-consuming and labor-intensive.

With these latest technological advances, we now have the ability to assemble near-complete genomes for any tree species, including conifers which account for 39% of the world’s forests but with giant genomes. Recently, based on PacBio and Hi-C technology, the 25.4 Gb chromosome-level assembly of Chinese pine (*Pinus tabuliformis*) was published^[168]^ which is 60 times larger than that of the first sequenced *P. trichocarpa* genome and represents a new milestone in the development of forest tree genomics.

Despite the recent progress, forest trees comprise an estimated ~73,000 species globally^[169]^. Thus the decoded genomes of 357 tree species merely represent a very small portion of diverse forest tree species. Moreover, there are still great challenges for the haplotype-solved assembly of complex genomes, especially for autopolyploid genomes owing to the high similarity of their subgenomes^[170]^. Although several freely available pipelines including TrioCanu^[171]^ , ALLHiC^[172]^, and FALCON-Phase^[173]^ have been developed for plant genomes assembled as chromosome-scale haplotypes, only four autoploid genomes, i.e., the sugarcane *Saccharum spontaneum* genomes (2n = 8x = 64, 2n = 4x = 40)^[174]^, the cultivated alfalfa genome (2n = 4x = 32)^[170]^, and tetraploid potato^[175]^ were *de novo* assembled at the chromosome level.

In addition to genome sequencing and assembly, the annotation of gene space has also received more attention. Recent RNA-seq studies hint that the transcriptomes are often substantially underestimated, even in the extensively studied important model organisms like Arabidopsis^[176]^ and rat (*Rattus norvegicus*)^[177]^. Large-scale RNA-seq data of at least a few hundred samples of different tissues conditions can provide direct transcript resources and enable an unbiased gene space annotation with high resolution^[168]^. However, obtaining diverse samples including different tissues and various induction conditions for RNA-seq analysis remains a labor-intensive challenge.

As more complete and accurate reference genomes become available, comparative genomics is rising. Due to newly developed sequencing technologies and updated assembly tools for producing longer reads and reducing assembly errors, many previous evolutionary findings are also worth re-examining. As all earlier draft released gymnosperm genomes reported a much older LTR outbreaks time which were later found to be overestimated by fragmented and less accurate assembly^[178]^. In addition, as more genomes of related species become available, pangenome-oriented studies are imminent.

## Perennial growth and seasonality regulation

Boreal and temperate climates are characterized with annual alterations of seasons that are favorable (spring and summer) and unfavorable or prohibitive (fall and winter) to growth. To survive the freezing and dehydration stress during fall/winter months, woody perennials from temperate latitudes temporarily suspend growth and protect the shoot apical meristem and the subtending leaf primordia in a specialized organ, known as the bud. The morphological, physiological and developmental processes taking place during this period are known collectively as dormancy.

Significant progress has been made in understanding the different stages of dormancy including the transitions from, and to, active growth. Here we focus only on the genomics aspects and prospects in forest trees. The molecular mechanisms regulating dormancy in fruit trees and vines, have been well reviewed^[179−187]^.

Although genomics attempts to study dormancy-related traits in forest trees were initiated *via* sequencing expressed sequence libraries (EST)^[188]^, it burgeoned only after the sequencing of the first tree genome in 2006^[162]^ and subsequently other forest tree genomes^[189−193]^ when these studies were scaled to the genome-wide level. Most prominently, availability of genome sequences, enabled for the first-time, a glimpse at the gene level landscapes of the QTL associated with dormancy^[194]^. Candidate genes found within the intervals were consistent with the molecular studies and pointing to genes encoding light receptors, circadian clock oscillators and regulators of the flowering time genes. In addition to QTL methods, the progress in genome sequencing spurred development of entirely new approaches known as association genetics and bringing the mapping resolution to the nucleotide level (e.g., single nucleotide polymorphism (SNP))^[195]^. Similar to the QTL studies, these approaches brought validation to the molecular studies and also identified new regulators of unknown functions^[195]^.

Perhaps the most wide-spread application of genomics approaches to dissection of dormancy traits is the characterization of transcriptomes associated with different stages. These were first done using microarrays and more recently by employing the more advanced RNA-seq technology^[196−204]^. Because of the advanced genomics tools and early adoption as a model for forest tree biology, many of the early and major transcriptomic characterization of dormancy were undertaken in poplar^[196,201,205,206]^. These studies have been expanded into many forest trees including angiosperms and gymnosperms and in different continents including North America, Europe and Asia^[196−204]^. These studies, when focusing on transcription factors, have employed chromatin immunoprecipitation (ChIP) to identify the direct targets among the many differentially expressed genes^[207,208]^. This has allowed insights into the hierarchical structure of the underlying networks and has led to discovery of new key regulatory factors. For example, transcription profiling of transgene-modified for the Early Bud Break 1 (EBB1) transcription factor of the AP2/ERF family has led to the discovery of Short Vegetative Phase-like (SVL) transcription factor^[206]^. This was later found to be another key regulator of bud-break and other dormancy stages^[207,208]^. However, although the above referenced studies only employed ChIP, the use of ChIP-seq should further expand the horizon of studies in this area and realize greater potential.

Role of epigenetics in regulation of dormancy traits is often speculated but rarely studied. Advances of epigenomics technologies could potentially address this void, including characterization of DNA methylation *via* bisulfide sequencing, posttranslational modifications of histones using ChIP-seq and sequencing of small/non-coding (nc) RNA libraries. However, these approaches have been rarely applied to dormancy traits. A notable exception is a study looking at the role of the DEMETER DNA demethylase on the genome-wide methylation pattern during the acquisition of growth competence after chilling and its correlation with transcriptomic changes^[209]^. This study pointed to key genes and processes affected during reactivation of growth including key regulators of meristem activity.

Methylation and acetylation marks at specific amino acids of the histone proteins are important cues regulating gene expression, and their effects can be studied via ChIP-seq approaches employing antibodies binding specifically to these modifications. Although the histone marks at individual gene loci in forest trees and mostly fruit trees have been studied^[210,211]^, a genome-wide map through different stages is still unavailable. Such maps combined with studies on methylation patterns as referred above can provide significant insights into the role of chromatin modification in regulating dormancy.

Small RNAs and ncRNAs have been recently linked to regulation of many processes, including vernalization^[212]^, a process suggested to be regulated in a similar fashion as dormancy^[182]^. Roles of these regulatory RNAs and their links to chromatin regulation are severely understudied in forest trees. miRNAs, a class of small regulatory RNAs have been linked to the ‘memory’ spruce embryos retain about the temperatures they experience during their development^[213]^. The temperatures at the time of embryo development can change phenological traits in one generation and these changes are stable over many years. For example, high temperatures during embryo development lead to later growth cessation and cold acclimation. It was speculated that epigenetic mechanisms are responsible and allow a high level of plasticity and adaptation that does not require lengthy cycles of natural selection. Sequencing of microRNA libraries have found correlation of the abundance of these molecules with different stages of this 'memory' acquisition^[214−216]^.

As genomics technologies become more advanced and affordable, there will be an even further increase in amount of genomics data. Although very useful, this data at the moment is largely descriptive. Development of advanced machine learning and artificial intelligence algorithms that can take advantage of this large data and provide insights into the causality, hierarchy and the regulatory landscape of the underlying mechanisms are needed.

Application and integration of multi-genomics and multi-omics approaches are required to provide system level knowledge of the underlying processes, however, these studies are still limited^[201]^. Conde et al.^[209]^ compared the genes with differential methylation pattern and changed expression to the gene set that was found with polymorphisms linked to bud-break^[195]^. Incorporation of such systems-level approaches spanning different regulatory and omics levels can bring novel insights and applications.

## Quantitative trait loci and association studies in forest trees

In order to design optimal early selection methods and breeding strategies, it is imperative to dissect the genetic basis and identify favorable alleles that underlie economically important and ecologically relevant traits. Most forest trees, unlike annual plants such as *Arabidopsis* and rice that would allow the use of reverse-genetic approaches to detect underlying genes, lack visible large collections of mutations of quantitatively inherited traits. Therefore, forward-genetic approaches such as QTLs and linkage disequilibrium (LD)-based association studies (AS) have been developed for identifying and dissecting quantitative traits in forest trees^[217]^. QTLs and AS have been broadly performed on traits of interest in numerous tree species, including growth and yield, wood properties, resistance to biotic and tolerance to abiotic stresses, and adaptive traits^[217,218]^, to promote the progress of molecular marker-assisted selection (MAS) breeding in trees^[219]^.

Due to the long juvenile periods and high heterozygosity of most forest trees, QTL mapping usually employs F_1_ full- or half-sib progenies. The availability of two-way pseudo-testcross strategy has promoted the construction of linkage maps in major timber species, but the limited resolution of these maps may not warrant a successful positional cloning of QTLs. Therefore, the use of high-throughput sequencing technologies can improve the accuracy and resolution of genetic maps, and allows the validation of the quality of scaffold anchoring and whole-genome assembly in trees^[220]^. Researchers developed several practical mapping algorithms to improve the speed and reliability of genetic distance estimation, which might alleviate the computing complexity and burden caused by map marker redundancy^[221,222]^. Currently, the bin mapping strategy^[223]^ has become the method of choice to construct dense genetic linkage maps.

QTL mapping has been conducted on a large scale in all major tree species groups for more than two decades. The classic strategies for QTL analyses include bulk segregation analysis (BSA), interval mapping and multiple interval mapping^[224,225]^. Currently, ultrahigh-density genetic maps based QTLs mapping in combination with systems genetic methods has provided the possibility of fine mapping and identifying genes in trees^[226,227]^. For instance, an integrated linkage-LD mapping was developed to improve the resolution and effect sizes of dynamic QTLs during stem growth in *P. tomentosa*^[228,229]^. The mapping noise caused by heterozygous genetic background can be greatly reduced by constructing haplotype modules based on linkage information and employing haplotype-based AS in QTL intervals^[219]^. In addition, genetic dissection of the segregation distortion of allelic variations is regarded as a key question of fine mapping and will likely be used in breeding in forest trees.

Forest trees are mostly undomesticated populations with wide geographical distribution, high maintenance of genetic variation, and low population differentiation, and thus are ideal systems for conducting AS and MAS breeding. Since Porth et al. conducted the first genome-wide association study (GWAS) for wood chemistry traits in black cottonwood (*P. trichocarpa*)^[230]^, hundreds of genetic marker loci have been found significantly associated with wood chemical composition and ultrastructural traits in *Populus*^[231,232]^, *Eucalyptus*^[217,233]^, *Pinus*^[234]^, and *Picea*^[235]^. Next-generation sequencing (NGS) has enabled more types of genetic variation such as insertion/deletions (InDels), structural variation (SV), and copy number variants (CNV) to be applied to GWAS to solve the problem of 'missing heritability'. For example, InDels can explain 14% of average phenotypic variance in growth and wood property-related traits in *P. tomentosa*^[236]^, and haplotype-based association analysis of multiple variation loci was also performed in *Populus*^[219]^ and *Eucalyptus*^[237]^. In addition, by integrating heterogeneous phenotypic data from different ages, loci, and pedigrees in 120 field experiments of 483,424 progenies of Norway spruce, the accuracy of GWAS phenotypic values were largely improved^[235]^. In recent years, the analytical strategy combining GWAS with multiple omics data has been applied widely in the investigation of the genetic architecture of wood variation^[228,238,239]^. Various new methods and strategies have been gradually applied to GWAS to improve the analytical accuracy, such as the development of high-throughput phenotyping techniques^[240]^, and to the detection of rare allelic and major structural variation^[217,241]^.

In boreal and temperate ecosystems, the adaptation of perennial plants to their surroundings is the major cue to regulate the seasonally synchronous annual growth cycle^[207]^, particularly, the key factors for the adaptation to changing conditions and environmental stressors over generations and the maintenance of standing genetic variation^[242,243]^. The application of environmental association analysis (EAA) allows the detection of the candidate genes involved in the environmental adaptation that results from populations across environmental gradients^[244]^. Many adaptive genetic variations have been determined in diverse and widespread woody plant genera. For example, a locus centered on *PtFT2* was identified using fitting latent factor mixed models (LFMM) in *Populus*, whose allele frequencies displayed a strong clinical pattern in latitude and had major effects on bud set^[245]^. In the *Quercus* range-wide model, six candidate SNPs were explored among the strongest environment-associated SNPs, offering robust evidence for local adaptation at multiple spatial scales^[246]^. These studies have determined the functional genes involved in the local adaptation that can be regarded as a gain or maintenance of divergent selection associated with complex environmental variables, and could potentially be utilized in ecological molecular breeding.

In reent years, our knowledge on the roles of epigenetic variation in diverse populations that underlie phenotypic variation has been growing. The rise of epigenetic quantitative trait loci (QTL^epi^) mapping and epigenome-wide association studies (EWAS) allow the detection of causal QTLs^epi^ and candidate genes, to overcome the insufficient effect of genetic dissection in explaining the 'missing heritability'. The first high-density linkage epigenetic map in tree species was constructed in* P. tomentosa*, and the QTLs^epi^ that control growth and wood property traits were detected^[247]^. This study may serve as a paradigm for making headway in the systematic mapping of complex traits in trees. Likewise, EWAS has deepened epigenetics variation research in trait regulation^[248,249]^. The discovery of the *Bad karma* locus in somaclonal variants of oil palms is one of the more prominent EWAS examples^[250]^. DNA methylation variation associated with climate gradients is also important for phenotypic plasticity and their ecological adaptation of forest trees^[251]^. The role of CG methylation in adaptation to climate and spatial variation was approved in natural oak populations^[252]^. Recently, DNA methylation level was reported to be affected by genetic variation cues. Trait-associated differentially methylated regions (DMRs) were reported to show evidence of chromatin interactions, enhancer activities^[253]^ or in linkage^[248]^ disequilibrium (LD) with nearby SNPs^[254,255]^. The interaction between DNA and methylation variations remains to be further investigated. The integrated linkage-LD mapping method using F1, F2, and backcross1 (BC1) populations will help to disentangle the causal methylation loci or regions that are responsible for heritable morphological variation from parents to progenies^[256]^. This will specifically reveal the contribution of allelic variants and overcome the inherent limitations of QTL^epi^ mapping.

'Missing heritability' has occurred in many QTL studies of tree species due to a variety of reasons that include, but are not limited to, small sample sizes, rare alleles, allelic heterogeneity, and epistasis. The genetic contribution of rare functional variants with high proportions of low-frequency genetic polymorphisms in forest trees remains unexplored^[232,234]^. Future studies on rare alleles in genetically diverse forest populations may perform experimental segregating validation in family-populations, and also improve detection efficiency using new statistical models and methods, such as the sequence-based kernel association test (SKAT)^[221]^, regional genetic mapping (RHM)^[218]^ and haplotype association analysis^[235]^. We are increasingly capable to address questions through a combination of alleles and haplotypes for traits of interest. However, functional interpretation and annotation of loci is still a critical challenge, especially as the majority of susceptible loci are located in non-coding or intergenic regions. The pleiotropic effects of genetic epistasis on gene expression, metabolites, and growth can explain the biological regulatory mechanisms beneath the statistical associations. Systems genetic approaches can help provide a comprehensive understanding of quantitative traits and enable the molecular design of new cultivars.

For the quantitative trait loci and association studies in forest trees, we expect that advances in systems genetics, including dense genetic linkage mapping, GWAS with multiple types of genetic variations, EAA, QTL^epi ^mapping, EWAS, and dissection of rare alleles, will promote data integration, upgradation and renovation, and provide a more powerful breeding guiding system for forest trees. The advent of precision genome editing such as CRISPR system in either genetic or epigenetic backgrounds will validate the causality of mutual exclusion (or heritable covariation) of traits and linkage drag, and will be an ideal method for the development of superior cultivars of forest trees.

## Genetic diversity and climate adaptation

Genetic diversity in forest trees is the foundation supporting their evolutionary potential in future generations. Populations with low levels of standing genetic diversity are predicted to have reduced responses to selection^[257]^, which could lead to increased risks of population decline or extinction under rapid environmental change. The genetic reservoir in a species is shaped by both stochastic neutral demographic events and natural selection over the course of species' evolutionary history. Species with wide distribution ranges often show a linear relationship between population genetic distances and geographical distances, a pattern defined as isolation by distance (IBD)^[258]^, which reflects decreasing rates of gene flow between more distantly located populations. IBD causes allele frequency clines among populations. Additionally, repeated founder events along species' migration routes can produce discrete genetic clusters and further amplify IBD^[259,260]^. In species that are widely distributed over heterogeneous landscapes, locally adapted populations are expected to harbor genetic compositions selected by the local environments. Gene flow among locally adapted populations is limited by selection because of lower fitness of immigrants, a process defined as isolation by environment (IBE)^[261,262]^. The consequences of these dispersal-demographic factors and selective forces are not mutually exclusive, and they often act together in generating population differentiation in natural systems^[259,261,263,264]^. Dissecting the separate contributions of these neutral and selective processes to population diversity is thus the first step towards a mechanistic understanding about the distribution of genetic diversity across a landscape.

Quantifying genetic variation in forest tree populations has been transformed by high throughput sequencing technologies in recent years, and transitioned from limited organellar and nuclear SSR markers to genome-wide scans. A variety of genome-wide genotyping methods, such as restriction-site associated DNA sequencing, genotyping-by-sequencing, whole exome capture sequencing, SNP array and resequencing, have provided improved spatial and temporal resolution of evolutionary dynamics of major forest tree species^[265−271]^. However, defining the genetic basis of local adaptation is not a straightforward task due to the complex biological processes involved. The detection of adaptive variation from genome-wide data is often approached by using outlier detection, association of allele frequencies with environmental variables (GEA), and evaluation of the contribution of IBD and IBE to population differentiation using redundancy analysis (RDA). By constraining either geography or environment in partial RDA models, the independent contributions of the two factors can be quantified. One pattern emerging from conifer species is that environment alone explains less than 10% of the allele frequency shifts among populations at all genomic SNPs, however this portion can go up to ~20% at GEA and *F*_ST_ outlier SNPs, although still leaving a large portion of the genetic diversity due to joint effects of IBE and IBD^[259,263,269,271,272]^. The strong confounding effect of IBE and IBD in natural populations makes disentangling adaptive variations among loci from the geographic relationships difficult^[273]^.

When both phenotypic and genomic data are available in parallel, phenotype-genotype associations can be evaluated using genome-wide association studies (GWAS), which offers a possibility of identifying the genetic causes of phenotypic variation. GWAS have been successful in pedigree materials, but association mapping in natural populations often end up with weak predictive powers of causal loci due to the confounding factors mentioned above and complex genetic interactions for polygenic traits^[274,275]^. Hall et al.^[269]^ illustrates this problem in their study on frost hardiness variation among Scots pine populations. They analyzed genotype–phenotype associations across 10 000 SNPs, and found a high marker-estimated heritability of the hardiness variation (0.56), illustrating the ability of genomic SNPs to capture the genetic variation in the trait. However, few loci appeared to have identifiable effects on the trait. The promising message from these studies is that as long as neutral-processes causing population structure are properly controlled and false positive rates are set at a reasonable level, GEA and GWAS can provide valuable information about adaptation dynamics across environmental gradients.

**Figure 3:**
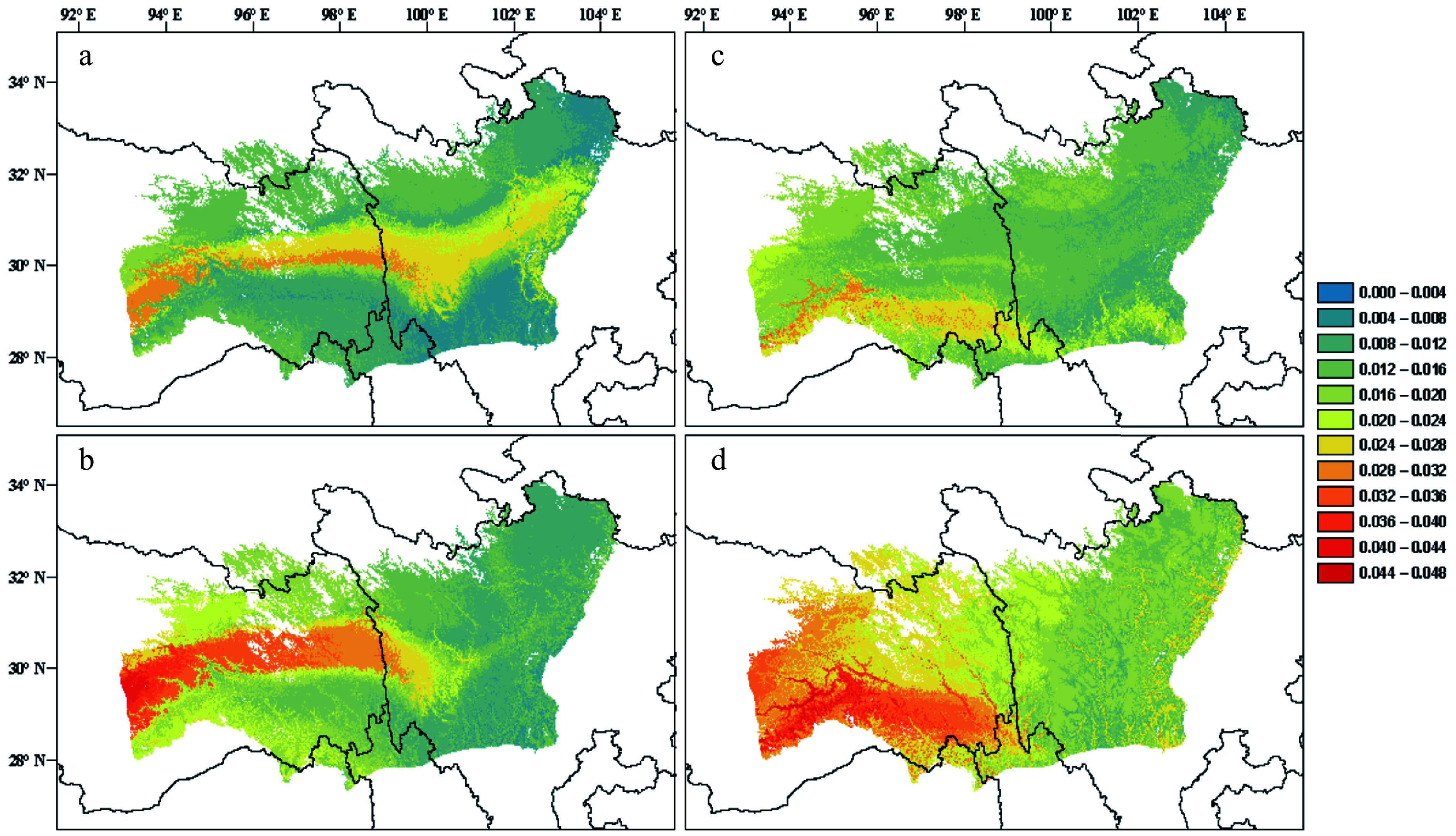
(a), (b) Predictions of genomic offset to future climate change in *Pinus densata* using a full set of 47,612 SNPs in exome sequences. (c), (d) Subset of 2,025 significant GEA SNPs. (a) and (c) reflect scenario representative concentration pathway (RCP) 2.6 2070; (b) and (d) reflect scenario RCP 8.5 2070. Red and blue indicate high and low genomic offset, respectively (Adapted from Zhao et al.^[259]^).

Knowledge about genetic adaptations across landscapes is informative for predicting the degree of genetic offset of current populations to future conditions^[276]^. The offset is often presented as a genetic distance between the extant and required genomic compositions for matching a future condition, assuming the current genotype-environment relationships in local populations are at equilibrium^[276,277]^. Gradient forest (GF), a machine learning algorithm, has been increasingly used to characterize GEA and to predict regions in a distribution range that are most vulnerable to climate change^[259,276,278]^. Offset projections can be simulated under different climate change, migration and gene flow scenarios to gain a preliminary assessment of potential actions needed to track changing conditions. Although a very attractive approach, many assumptions in offset simulations are difficult to meet with certainty, e.g., defining adaptive and neutral genetic variation and the position of local populations on the adaptive landscape^[259,279]^. As illustrated in Fig. 3, genomic offset projections using different SNP sets convey different biological implications, thus may not align well with each other. A future improvement is to incorporate fitness effect estimates of allele frequency changes into GF models of species distribution shifts^[279]^. In this regard, long-term large-scale provenance trials remain irreplaceable even in the genomic era for model validation of environmental responses of forest trees.

To conclude, rapid climate changes represent a challenge for many forest tree species. Due to their long generation time and the often-limited seed dispersal, genetic adaptation in forest populations lags behind environmental change. Knowledge about genetic diversity and genetic adaptation in natural populations is therefore essential for sustainable management of forest ecosystems. Applications of this research include the development of guidelines for assisted gene flow^[280]^, breeding zones, seed transfer for forest restoration, and conservation of endangered species and populations.

## Genotyping efforts and genomic selection in forest trees

Intensively managed forests are essential sources of fiber, biomass, pulp/paper, and timber in many countries worldwide^[281]^. Managed forests provide raw materials to industry sustainably while reducing the pressure on natural forests. For example, in the southern United States, about 20% of forestland is managed for softwood production, yet the region produces more than 55% of the timber^[282]^. Managed forests are significant sources of carbon storage, and they help mitigate greenhouse gas emissions. Forest tree breeding is an essential part of growing woody biomass sustainably. Moreover, genetics and breeding are usually the only way to improve the forests for biotic (pests and pathogens) and abiotic (air pollution, climate-change-related factors) stresses.

Despite the critical role of forest tree breeding for planted forests, forest tree breeding has not received the needed resources and support as crop and animal breeding programs. Forest tree breeding is still in its early stages. Tree breeding activities in some developing countries (e.g., Sweden, Finland, and the USA) started in the 1950s; however, modern tree breeding did not start in many countries until the 1980s. Even though the breeding of the major conifers started more than 60 years ago, the progress has been limited. The slow progress of tree breeding is due to the lack of resources, as tree breeding has not been considered as important as crops. Other reasons are biological. Forest trees, especially conifers, take many years to mature and flower. The flowering is not frequent for many northern latitude conifer species. Field testing takes years, sometimes a decade, to collect data and make selection decisions. Shifting program priority and scientist turnover, may also disrupt the long breeding effort.

**Figure 4:**
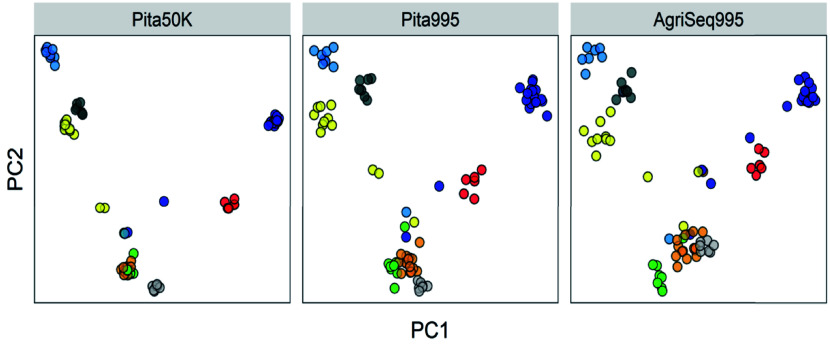
Clustering eight *Pinus taeda* full-sib families (colored circles) based on three different SNP marker sets. AgriSeq Targeted GBS panel (on the right) clustered the full-sib families similar to the same SNP markers selected from the *Pinus taeda* SNP array (in the middle). Trees not clustered are likely pedigree errors or the half-sibs (sharing one parent of another full-sib family).

Advances in DNA sequencing technologies have impacted plant and animal breeding since 2008. Breeders are now equipped with thousands of single nucleotides polymorphic (SNP) markers to fundamentally change plant and animal breeding. SNP markers are promising to make selection of breeding germplasm at a juvenile stage, a process called genomic selection. Genomic selection has doubled genetic gain per unit of time in cattle breeding^[283]^. The impact of genomic selection and other applications of DNA markers in forest tree breeding is expected to be even higher because of longer breeding cycles of forest trees^[284]^. SNP arrays and genotyping-by-sequencing are the most common genotyping platforms for forest trees^[285−288]^. Forest tree breeders have shown a great interest in genomic selection (GS) to reduce the long breeding cycles^[289,290]^. Several studies on cost-benefit analysis suggested that GS selection is feasible^[291,292]^. However, the progress in the implementation of GS in forest tree breeding is still behind animal and major crop breeding. High genotyping cost per sample is one of the limiting factors. Developing more cost-efficient genotyping platforms is still active research^[293]^. For example, at North Carolina State University Tree Improvement Program, AgriSeq Targeted GBS panel designed by Thermo Fisher Scientific is promising for some molecular applications in *Pinus taeda* breeding (unpublished). The panel is based on a small subset of SNP markers selected from *Pinus taeda* SNP array Pita50K^[285]^. The quality control statistics are comparable to the SNP array. For example, the sample call rate is 86%, and sample uniformity is 89%. The following plot shows how the AgriSeq Targeted GBS panel clustered eight full-sib families compared to the Pita50K SNP array and a subset of array markers amplified for AgriSeq panel (Pita995) (Fig. 4).

A routine application of GS requires a reliable and cost-efficient genotyping platform. Although the cost of DNA sequencing has dropped sharply since 2008, the cost per sample using SNP arrays is still somewhat high, especially for many developing countries. Targeted sequencing-based methods are promising genotyping platforms to reduce the cost. Research in developing cost-efficient genotyping platforms will continue to be an important subject. Developing and updating high-quality reference genomes of forest trees needs to be prioritized. Improved reference genomes can greatly enhance marker discovery, annotation, haplotype construction, imput of missing genotypes, and QTL discovery. The current reference genomes of forest trees, especially conifers, are highly fragmented^[294]^. To be useful for many applications, they need substantial improvement using the latest long-read sequencing technologies, such as PacBio sequencing.

## Computational biology approaches in identifying genes controlling biological processes and complex traits in forest genomics-based research

The advent of microarray and RNA sequencing (RNA-seq) technologies has generated an enormous high-throughput gene expression data, which are usually analyzed with various statistical methods, leading to differentially expressed genes (DEGs). Following that, DEGs-based enrichment analyses including gene ontology (GO), protein domain and pathway enrichment analyses can be performed using Fisher Exact Test or hypergeometric distribution to reveal enriched moieties or entities^[295]^. These analyses are useful but provide little information about the organization of the genes in regulatory, collaborative, or interactive networks, which are essential for discovering underlying and novel molecular mechanisms essential for advancing our understanding. To build gene regulatory networks, many algorithms based on various mechanistic and statistical modelings have been developed. These methods can be, by and large, classified into two categories: dynamic and static methods. Dynamic methods include differential equation^[296]^, finite state^[297]^, dynamic Bayesian^[298]^, control logic^[299]^, Boolean^[300]^, and stochastic networks^[301]^, which requires true time-course data with small intervals. This kind of data can be readily generated for prokaryotes and unicellular organisms like yeast. Static methods, which do not necessarily require time-course data, are represented by graphical Gaussian models (GGM)^[302]^, mutual information based relevance networks^[303]^, Algorithm for the Reconstruction of Accurate Cellular Networks (ARACNE)^[304]^, Context Likelihood of Relatedness (CLR)^[305]^, C3NET^[306]^, Mutual Information 3 (MI3)^[307]^, and Bayesian probabilistic network^[299]^. Although static methods can also be used to analyze time-course data, they do not take temporal dependance and causality into consideration, which may cause some information loss. Although these dynamic and static methods can broaden our understanding of underlying gene regulatory networks (GRNs), they are not designed and tailored to generate inferences about the hierarchical architecture of the GRNs.

**Figure 5:**
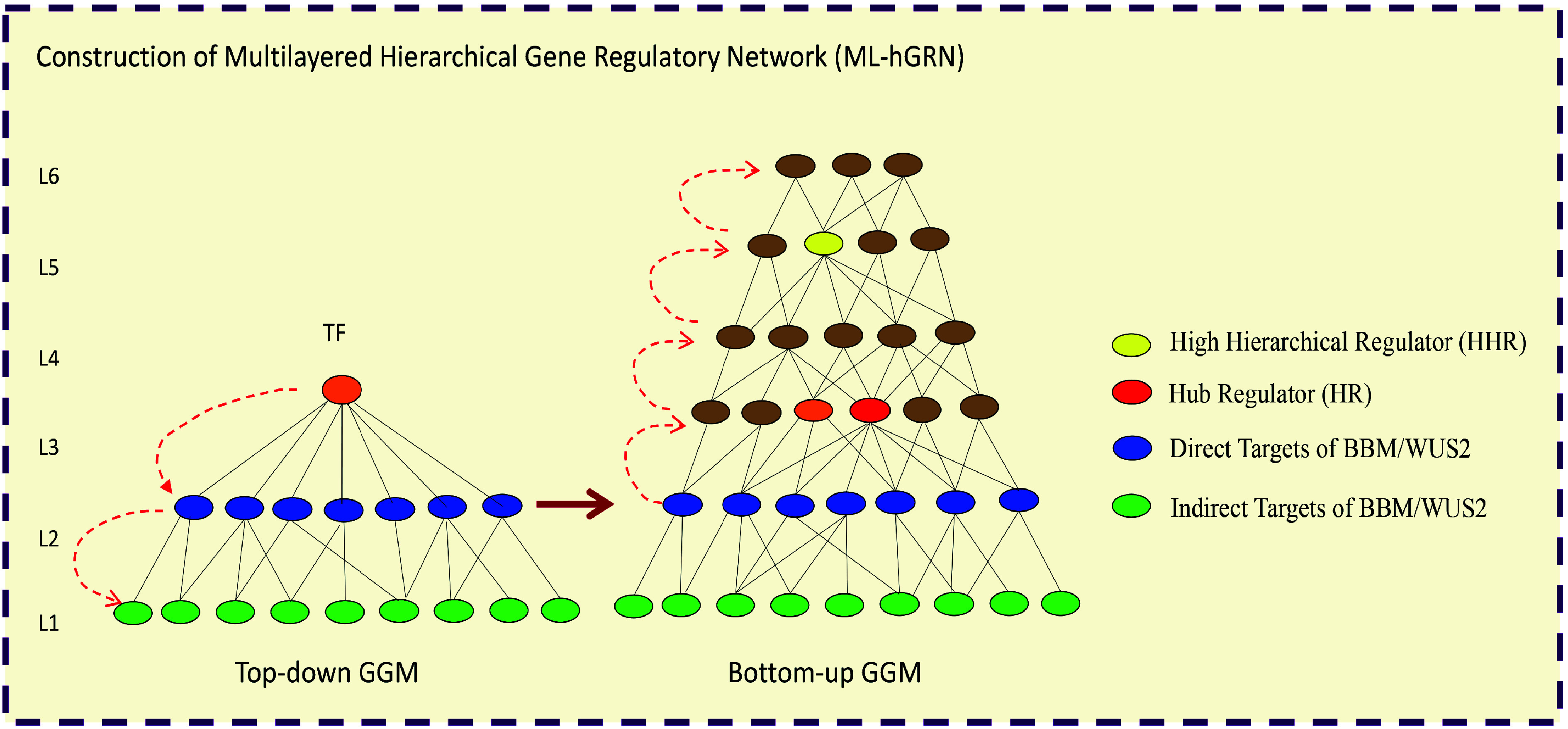
Illustration of how to construct a multilayered hierarchical gene regulatory network (ML-hGRN) to encompass a given transcription factor (TF) using Top-down GGM and Bottom-up GGM Algorithms synergistically.

Recently, algorithms for construction of hierarchical GRNs from high-throughput gene expression have been established. These approaches can be used to infer the multilayered hierarchical network mediated by a transcription factor. For example, Top-down GGM Algorithm^[308]^ for constructing a multilayered gene regulatory network mediated (ML-hGRN) by a regulatory gene like a transcription factor (TF). Top-down GGM algorithm has been employed to construct a ML-hGRN mediated by PtrSND1^[308]^, PuHox52^[309]^, PpnGRF5^[310]^, Ptr-miRNA319a^[311]^ and BplERF1^[312]^. The method is especially valuable when being used in conjunction with the perturbation of a regulator gene/TF followed by RNA-seq assay; the method has been shown to capacitate the separation of direct from indirect target genes with high accuracy^[308]^. On the contrary, Bottom-up GGM Algorithm^[311,313]^ and Backward Elimination Random Forest Algorithm^[314]^ have been developed for inferring ML-hGRN operating above a biological process or a pathway. From the ML-hGRN built with Top-down GGM Algorithm, the hierarchical regulators at different levels can be identified. Thus far, Bottom-up GGM Algorithm has been used to identify Ptr-miRNA319a^[311]^ functioning above lignin polymerization pathway, and PuMYB40 and PuWRKY75 functioning as high hierarchical regulators above the biological process of low phosphorus (LP)-mediated adventitious root (AR) formation. Bottom-up GGM Algorithm and Bottom-up GGM can be used synergistically to infer the ML-hGRN encompassing a TF^[315]^, as shown in Fig. 5.

One of the central tasks of genomics is to identify the genes regulating a biological process or a complex trait directly from high-throughput transcriptomic data. To do this, the high-throughput data needs to be generated from a tissue or an organ in which a trait of interest is under a transition. This kind of transcriptome is easy to produce. However, until now the genes controlling a trait have been primarily identified through linkage mapping and genome-wide association studies, which is labor-intensive and time-consuming, and may not guarantee a success in most cases where pleiotropy is dominant. Nevertheless, a new theory has been proposed for identifying the genes regulating a biological process or a complex trait from transcriptomes, which assumes that the genes that regulate a biological process or a complex trait often collaborate throughout the time period during which the biological process or the complex train is under a transition^[316,317]^. Based on this theory, a method was developed for building the collaborative gene regulatory network which was then decomposed into many small subnetworks, each containing regulatory genes collaboratively controlling a biological process or a trait. The method has been applied to multiple transcriptomic data sets in multiple species and the results showed that regulatory genes governing a biological process or a complex trait were captured in almost every case.

Recently, a method for joint construction of multiple gene GRNs using gene expression data from multiple tissues or conditions was developed^[318]^. The method allows for identifying the common regulators across multiple tissues/conditions and unique regulators peculiar to just one tissue. In addition to this, integrative analysis of spatial transcriptome, single-cell RNA-seq and RNA-seq data has emerged^[25,91]^, which indicates a new era for interpretation of spatiotemporal data yielded from development and differentiation processes has arrived. Obviously, more advanced software pipelines and tools for analyzing multi-sourced data or real spatiotemporal data are needed.

## Concluding remarks

It becomes increasingly clear that high-throughput sequencing technologies, together with the newly available scRNA-seq, CRISPR-mediated genome editing, CRISPR-mediated upregulation/downregulation, spatial transcriptome and advanced bioinformatics analysis technologies have provided unprecedented opportunities to leverage the important and unique development and differentiation-related issues of forest trees. The large amounts of data will help to study molecular mechanisms of tree growth and development as well as genome-assisted forest tree breeding. Future research in forest genomics is being refocused on some important developmental, evolutionary, and adaptative problems, for example, stem cell entity maintenance, tissue, organ and architecture formation, secondary growth, transformation recalcitrance, nitrogen fixing, perennial growth and seasonality regulation, genome evolution, genetic diversity and climate changes, QTL mapping and genomic selection, and regulatory mechanisms underlying various complex traits. Forest genomics research is moving towards more interdisciplinary endeavors. With effective collaboration among global researchers from different disciplines, we can optimistically foresee the even greater, and faster advancement in forest genomics and systems biology, which will maximize the potential of developing 'desirable, customer-designed forest trees', for improving our environment, and fighting climate change.
